# Practical Notes and Formulæ

**Published:** 1879-09-20

**Authors:** 


### PRACTICAL NOTES AND FORMULÆ.

**Dyspepsia.**—

℞ Ext. nux vomica........................................ gr. iij.

Make 24 pills.

Dose, one pill three times a day.

℞ Carb. soda........................................ ℥j.

Aromat. spts. ammonia, essence menth pip........aa ℥j.

Sulphite soda........................................ ʒij.

Aqua dest........................................ Ojss. M

Dose, a teaspoonful before meals, for acid dyspepsia.

℞ Comp. tinc. cinchona, tinc. hydrastis..................aa ℥ij.

Nitro-muriatic acid........................................ ʒj. M

Dose, a teaspoonful three times a day.

℞ Tinc. gentian, tinc. hydrastis..........................aa ℥ij.

Tinc. nux vomica........................................ ʒij.

Hydrocyanic acid dil........................................ gtt. xx. M

Dose, a teaspoonful before meals.

℞ Pulv. gentian, quassia..........................aa ʒiij.

Prunus virg........................................ ℥ij.

Cinnamon, bitter orange peel..................aa ʒj.

Alcohol........................................ Oj.

Water........................................ Oiss. M

Put in a covered vessel and steep two weeks.

Dose, a teaspoonful before each meal, which can be increased to a wine-glassful.

Three drops of dilute sulphuric acid in a wine-glass half full of water four times a day. For acid dyspepsia.

I have thus treated with unfailing success several hundreds of such cases, whether of dyspepsia pure and simple or complicating cardiac disease. Should be given without any admixture.—C. R. FLEURY, M.D.

℞ Taraxacin, hydrastin..................................each 30 gr.
Ext. nux vomica........................................ 5 gr.
Make 20 pills.

Dose, one pill three times a day. For dyspepsia with constipation. —W. W. N., in *N. Y. Eclec. Med. Journal.*

**Artificial Cataplasm.**—Volkhausen prepares cataplasms consisting of a piece of white thick felt paper which is saturated with a decoction of linseed. When intended to be used, the paper is dipped into hot water, swells considerably, is then applied, covered with caoutchouc paper, fastened with bandages or string, and allowed to remain for twelve hours before a new one needs to be applied.—*Amer. Journal of Pharmacy.*

**Topical Uses of Ergot.**—Dr. William C. Dabney has used ergotin in conjunctivitis, where the blood-vessels were enlarged and tortuous, with excellent results. The eye was frequently cleansed with warm water, and after each washing a few drops of the following solution were instilled :

℞ Ext. ergotæ........................................ gr. x.

Glycerinæ........................................fl ʒj.

Aquæ, ad........................................fl ℥j. M

In acute conjunctivitis, or where there is much intolerance of light, the result is not so satisfactory.

In pterygium, Dr. Dabney has also used ergot successfully, a solution of the strength mentioned being used three times a day, and the growth checked thereby. In pharyngitis a solution of Squibb's solid extract is useful, and in other pharyngeal affections the following formula has been found to do good :

℞ Ergotinæ........................................ gr. xx.

Tinc. iodini........................................fl ʒj.

Glycerinæ, ad........................................fl ℥j. M

To be applied to the pharynx freely twice a day with a camel's-hair brush.

In uterine troubles, particularly cervical metritis, the following suppositories may be used with advantage :

℞ Ergotinæ (seu ext. ergotæ)........................ gr. xx.
Ext. belladonnæ........................................ gr. ij.
Ol. theobromæ........................................ q. s.
Mix. Fiat in suppos. No. vi.

Insert into the vagina every night after the hot douche. In warm weather, a pledget of cotton saturated with the following solution may be inserted into the vagina every night after the hot douche :

℞ Ergotin. (seu ext. ergot. fld.)........................ ʒ ss
Ext. bellad........................................ gr. vi.
Aqua et glycerin........................................aa fl ℥ iv. M

—*Amer. Journal of the Med. Sciences.*

**Concentrated Solution of Salicylic Acid.**—I have found the following mixture an excellent method of administering salicylic acid.

℞ Acidi salicylic. pur........................................ ʒ i.

Sodii biboratis........................................ ʒ ss.

Glycerinæ........................................ q. s M

Mix acid and borax with glycerine two drachms and beat until dissolved, then add enough of the glycerine to make half an ounce. This can be diluted for administration with either glycerine, alcohol or water, to any degree required.—*Med. and Surg. Brief.*

**Chaulmoogra Oil.**—This oil is highly recommended in scrofula of children. The dose is six to fifteen drops to adults, three times daily, a short time after meals. The dose for infants is from two to three drops. The oil is best administered in cod-liver oil, or, when this cannot be taken, in glycerine or milk. It should at the same time be applied externally. Spices should not be eaten, but a fatty diet is recommended.

**Inhalation of Eucalyptus Oil.**—Dr. Mosler, of Greifswald, in Berliner Klin. Wochenschrift, strongly recommends oil of the leaves of eucalyptus, administered by inhalation, as a remedy for pharyngeal diphtheria. The strongest dose which he has given was according to the following formula :

| | | |
|---|---|---|
| ℞ | Oil of eucalyptus leaves........................ | 5 grammes. |
| | Rectified spirit.................................... | 75 " |
| | Distilled water..................................... | 170 " |

To be shaken and used for ten inhalations.

In this dose the medicine was inhaled four times daily, for ten or fifteen minutes each time, by a patient suffering from bronchitis and chronic laryngitis; it produced no troublesome effect, but acted as a powerful expectorant.

Another formula employed by him was :

| | | |
|---|---|---|
| ℞ | Oil of eucalyptus leaves............................ | 2 grammes. |
| | Rectified spirit........................................ | 20 " |
| | Distilled water........................................ | 180 " |

For ten inhalations.

This was given with the best effect in a case of croupous pneumonia in the stage of defervescence, with residual infiltration of the right upper and middle lobes. It was inhaled four times without any bad effect. A still weaker pr paration :

| | | |
|---|---|---|
| ℞ | Eucalyptus oil.......................................... | 1.5 grammes. |
| | Spirit of wine.......................................... | 15 " |
| | Water...................................................... | 200 " |

Has been used by him in several cases of nasal and pharyngeal catarrh, and also in a case of acute pharyngitis accompanied by slight laryngitis, with good effect.

Dr. Mosler is engaged in further researches on the action of inhalation of eucalyptus oil in affections of the respiratory organs.—*British Medical Journal.*

**To Expel Ascarides.**—Dr. Guichon, of Paris, France, gives the following :

| | | |
|---|---|---|
| ℞ | Santonini pulveris............................................ | ʒj. |
| | Resinæ jalapæ................................................. | gr. ij. |
| | Chocolati......................................................... | ℥j. |
| | Mix and divide into 30 powders. | |

Give one in the morning on an empty stomach, to an infant two years old; two or three to older children. Also use :

| | | | |
|---|---|---|---|
| ℞ | Aloes barbadensis............................................ | ʒss. | |
| | Potassii carbonatis............................................ | gr. xv. | |
| | Decocti amyli.................................................. | ℥x. | M |

To be used as an injection in ascarides of the rectum.

**Enuresis in Children.**—Some recent British writers state that after numerous failures with all orthodox modes of treatment, it was found that cutting off meat in the diet was sufficient in many cases, to effect a rapid and permanent cure.—*N. Y. Med. and Surg. Reporter.*

**Treatment of Gonorrhœa.**—Having used every treatment I could get from different journals, and a great many sent to my drug store by numerous physicians, I have the right to select and advocate the one that has given me success in every instance, except with those who will not leave off their whisky, and success with that class oftener than failure. If I am advised with at the beginning of the trouble, I prescribe :

| | | |
|---|---|---|
| ℞ | Hoffman's anodyne........................................ | ℥ ij. |
| | Sweet spts. nitre........................................ | ℥ ij. |
| | Bal. cop........................................ | ℥ i.j |

Dose, eightty drops three times a day.

| | | |
|---|---|---|
| ℞ | Chlor. pot........................................ | 40 grains. |
| | Quinine........................................ | 16 " |
| | Arom. sulph. acid........................................ | 10 " |

Dissolve quinine in acid, mix in 8 ounces of dist. water and add chlorate pot.

Inject three times a day, urinating first.

I also order a mild saline cathartic every third day, and a teaspoonful of Battle & Co.'s Bromidia at bed-time. I find the bromidia to act better than any anodyne I ever used; it successfully prevents painful erections of the penis. After using the above treatment for five or six days, I then prescribe 10 grains of iodide pot. in a desertspoonful syrup sarsaparilla. It will cure all cases inside of four weeks. The iodide of pot. is a treatment of my own; I have never read of its being used in this disease. I attribute my success to the use of this drug.—S. E. Brown, M.D., in *Med. Brief.*

**Modified Dover's Powder.**—Dr. Tully's popular modification of Dover's powder is, according to Mr. Wood, of New Haven, prepared as follows:

| | | |
|---|---|---|
| ℞ | Morph. sulphatis........................................ | 1 part. |
| | Pulveris camph........................................ | 20 " |
| | Creta precipit........................................ | 20 " |
| | Pulv. glycyrrh........................................ | 20 " M |

This is the original formula of Dr. Tully.

According to the same authority, the "camphorated Dover's powder" of Dr. Eli Ives is as follows :

| | | |
|---|---|---|
| ℞ | Potass. bitartratis........................................ | 8 parts. |
| | Pulveris camph........................................ | 2 " |
| | Pulveris ipecac........................................ | 1 " |
| | Pulveris opii........................................ | 1 " |

**Cardiac Dyspnœa.**—Prof. See, of Paris, advocates the use of iodide of potassium in cases of continuous cardiac dyspnœa, either alone or combined with opium, digitalis or chloral, beginning with doses of 1¼ gram, and rising gradually to 2 or 3 grams, to be continued for some time. Opium is added, in doses from 10 to 15 centigrams, in order to counteract the effects of iodine; and chloral is useful in cases where digitalis is not tolerated. The prescription would then be as follows :

| | | |
|---|---|---|
| ℞ | Gum julep........................................ | 120 grams. |
| | Iodide of potassium........................................ | 2 " |
| | Hydrate of chloral........................................ | 4 " |

To be taken in quantity as required, every two hours during the day.

